# Hypophosphatasia: from birth to adulthood

**DOI:** 10.20945/2359-3997000000626

**Published:** 2023-05-29

**Authors:** Fernanda Salles Reis, Marise Lazaretti-Castro

**Affiliations:** 1 Universidade Federal de São Paulo Disciplina de Endocrinologia Departamento de Medicina São Paulo Brasil Departamento de Medicina, Disciplina de Endocrinologia, Universidade Federal de São Paulo (Unifesp), São Paulo, Brasil

**Keywords:** Hypophosphatasia, asfotase alfa, TNSALP mutation, alkaline phosphatase

## Abstract

Hypophosphatasia (HPP) is an inherited disease caused by a low activity of tissue-nonspecific alkaline phosphatase, a hydrolase that removes phosphate groups from many molecules. Decreased alkaline phosphatase activity leads to the accumulation of three main metabolites, *i.e.,* pyridoxal 5'-phosphate (PLP), inorganic pyrophosphate (PPi), and phosphoethanolamine. Impairment in PLP dephosphorylation induces seizures, while PPi accumulation inhibits bone mineralization. Clinically, HPP has a wide spectrum of presentations, ranging from neonatal death to an apparent lack of symptoms. This disease is classified into six subtypes according to the age at onset of first signs or symptoms. The clinical manifestations of the disease include rickets-like bone changes, bone demineralization, fragility fractures, reduced muscular strength, chest deformity, pulmonary hypoplasia, nephrolithiasis, nephrocalcinosis, and chondrocalcinosis. Treatment of HPP consists of enzyme replacement therapy. Before this therapy was approved, treatment was palliative and associated with high morbidity and mortality. Asfotase alfa has changed the prognosis of the disease by reducing bone deformity and improving bone mineralization, lung function, and muscle weakness, among other benefits. In adults, teriparatide and anti-sclerostin antibody have been used off-label in selected cases, demonstrating benefit in accelerating fracture healing and in concomitant treatment of osteoporosis. This review summarizes the main aspects of HPP and identifies the particularities of the disease in adult patients.

## INTRODUCTION

Hypophosphatasia (HPP), an inborn error of metabolism, is an inherited disease caused by a low activity of tissue-nonspecific alkaline phosphatase (TNSALP). The disease was first described in 1948 by John C. Rathbun in a patient with rickets, seizures, and reduced alkaline phosphatase (ALP) activity in serum and bone tissue (1). Four genes encode ALP, of which three are tissue-specific, *i.e.*, intestinal, placental, and germ cell (*ALPI*, *ALPP*, and *ALPPL2*, respectively). The *ALPL* or *TNSALP* gene, composed of 12 exons, encodes the TNSALP protein, which is present in various tissues, including bone, liver, and kidney (2). TNSALP encompasses a family of enzymatic isoforms that differ in physicochemical properties, rendering it multiple functions, including bone mineralization (2,3). Patients with elevated ALP levels due to hepatobiliary disease do not present increased levels of the bone isoform of the enzyme, while those with elevated ALP levels due to Paget's disease present only increased levels of the bone isoform (4).

### Epidemiology and genetics

The highest prevalence of HPP has been described in Canada, where the severe form of the disease occurs in 1:100,000 live births (5). The estimated prevalence rates of severe disease forms per live births are 1:300,000 in Europe and 1:300,000-500,000 in Japan (6-8). Brazilian data on HPP prevalence is currently not available.

More than 400 mutations in the *TNSALP* gene have been described to date, most of which comprise missense mutations (https://alplmutationdatabase.jku.at/). Inheritance of this gene can occur by autosomal dominant or recessive transmission, explaining the different degrees of severity of the disease (9). The most severe forms of HPP are associated with greater suppression of TNSALP activity and, in most cases, autosomal recessive inheritance (6,10). Mutations that slightly reduce TNSALP activity are associated with milder manifestations. The same mutation can lead to clinical manifestations of different severity if inherited in homozygosity or heterozygosity (11,12). Additionally, the genotype-phenotype correlation varies, and different phenotypes can occur in the same family (13).

### Pathophysiology

**Figure 1:**
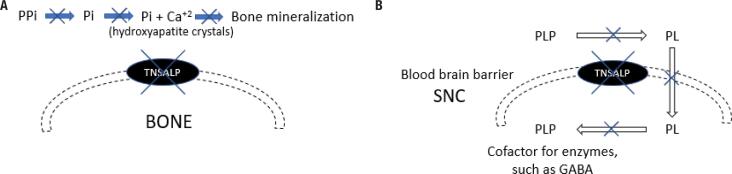
(**A**) Pathophysiology of bone demineralization in hypophosphatasia. (**B**) Pathophysiology of seizure in hypophosphatasia. **A.** Mineralization begins with the formation of hydroxyapatite crystals from calcium and phosphate (Pi). Inorganic pyrophosphate (PPi) inhibits the formation of hydroxyapatite crystals. Thus, it must be hydrolyzed by alkaline phosphatase (TNSALP) for mineralization to occur. With the reduced TNSALP activity in hypophosphatasia, PPi is not hydrolyzed, and bone mineralization is decreased. **B.** In the most severe cases of hypophosphatasia, pyridoxal 5'-phosphate (PLP) is not dephosphorylated effectively, and pyridoxal (PL) becomes unable to cross the blood-brain barrier and participate in the formation of gamma-aminobutyric acid (GABA). This leads to vitamin B6-dependent seizure.

The occurrence of HPP is due to a loss-of-function mutation in the *TNSALP* gene. The hydrolase ALP removes phosphate groups from several molecules, and its decreased activity leads to the accumulation of three main metabolites, *i.e.*, pyridoxal 5'-phosphate (PLP), inorganic pyrophosphate (PPi), and phosphoethanolamine (PEA). Impairment in PLP dephosphorylation may lead to seizures, while PPi accumulation inhibits bone mineralization. The consequences of PEA accumulation remain largely unknown (Figure 1) (14).

In bone, TNSALP is present on the cell surface of osteoblasts and hypertrophic chondrocytes, where it hydrolyzes PPi to inorganic phosphate (Pi), which in turn binds to calcium to form hydroxyapatite crystals. PPi is a potent inhibitor of bone mineralization; its concentrations increase in the absence of TNSALP, inhibiting the nucleation and growth of hydroxyapatite crystals (Figure 1A). This leads to bone hypomineralization, increased risk of fracture, and ectopic calcifications due to increased calcium x phosphorus (Pi or PPi) product in peripheral tissues (14-16).

Hypercalcemia and hypercalciuria may occur due to reduced use of calcium in bone mineralization, resulting in nephrocalcinosis and reduced levels of parathyroid hormone (PTH) (17,18).

Hyperphosphatemia is common in HPP and occurs even in cases with mild disease. The occurrence of hyperphosphatemia is apparently related to an increase in the maximum renal tubular threshold for phosphorus adjusted by the glomerular filtration rate (TmP/GFR) (19). The following three main mechanisms are suggested for this occurrence: (1) reduced TNSALP activity, which seems to facilitate the excretion of phosphorus by the kidneys; (2) excess PPi, which competes with Pi for a shared transport mechanism; and (3) change in concentration of phosphatonins, which have a phosphaturic effect. Low levels of fibroblast growth factor 7 (FGF7) and normal levels of fibroblast growth factor 23 (FGF23) and secreted frizzled-related protein 4 (sFRP4) have been observed in pediatric HPP (20). In severe cases, hyperphosphatemia may occur due to secondary hypoparathyroidism (19).

Notably, TNSALP works by removing the phosphate group from PLP, the active form of vitamin B6, enabling PLP to cross the plasma membrane. In the cytoplasm, pyridoxal is again phosphorylated and functions as a cofactor in several enzymatic reactions. In the most severe cases of HPP, PLP is not dephosphorylated effectively, becoming unable to cross the blood-brain barrier and participate in the formation of gamma-aminobutyric acid (GABA, an inhibitory neurotransmitter), leading to vitamin B6-dependent seizure (Figure 1B) (21,22).

Frequent manifestations of HPP include muscle weakness and pain, whose pathophysiology is poorly understood. Notably, TNSALP is also present in endothelial and neuronal cells and in the skeletal muscle (23).

### Clinical manifestations

The spectrum of clinical presentations of HPP varies widely, ranging from neonatal death to an apparent lack of symptoms. In the most severe forms of HPP, it is possible to detect during the prenatal period the occurrence of polyhydramnios, bone demineralization, shortening of long bones, fractures, and pulmonary hypoplasia (14). Manifestations of mild cases are often only identified with active questioning. In these cases, there may be loss of teeth occurring early in life or with an intact root, shorter stature compared with family members, delayed motor development, and fatigue (Table 1) (24).

**Table 1:** Clinical manifestations of hypophosphatasia

Clinical manifestations
**Skeletal:** rickets-like bone changes, bone demineralization, bone deformity, fragility fractures, pseudofractures, delayed bone healing, low stature, craniosynostosis
**Muscular:** reduced muscular strength, altered gait, and myalgia
**Neurological:** seizure, complications associated with craniosynostosis, and intracranial hypertension
**Respiratory:** chest deformity, pulmonary hypoplasia, frequent respiratory infections, tracheomalacia
**Renal:** nephrolithiasis, nephrocalcinosis, hyperphosphatemia, hypercalciuria
**Articular:** chondrocalcinosis, periarticular calcification, pseudogout
**Dental:** early tooth loss
**Other manifestations:** failure to thrive

Adapted from: Conti F. Hypophosphatasia: clinical manifestation and burden of disease in adult patients. Clin Cases Miner Bone Metab. 2017;14(2):230-4.

Other musculoskeletal manifestations of the disease include fragility fractures and bone deformities, rickets-like bone changes, reduced muscular strength, reduced walking distance, altered gait, and myalgia (24). Nephrocalcinosis and nephrolithiasis have also been described (17).

Neurological manifestations of HPP are associated with craniosynostosis, which is very common in the disease and in other forms of rickets (25). Craniosynostosis occurs due to early closure of cranial sutures. In addition to aesthetic impairment, craniosynostosis is associated with an increased risk of intracranial hypertension, which can lead to cognitive and vision impairment. Craniosynostosis usually occurs slowly and progressively, and symptoms of acute intracranial hypertension, such as headache, nausea, vomiting, and altered consciousness, are not usually present (26). The diagnosis of craniosynostosis is based on clinical presentation and imaging tests. Fundoscopic examination should be performed in all patients to evaluate for intracranial hypertension (25). After neurosurgical treatment, the sutures may again close prematurely, and further surgery may be necessary. Therefore, monitoring of intracranial hypertension is suggested until the skull reaches final growth (26).

Respiratory manifestations of HPP include chest deformity, rib fractures, pulmonary hypoplasia, muscle weakness, tracheomalacia, and frequent respiratory infections. Before treatment became available, HPP was considered fatal, with 50%-100% of the patients dying due to respiratory complications (14).

The main manifestations of HPP in adults are musculoskeletal complaints. The patient's history may include several orthopedic surgeries and nontraumatic and difficult-to-heal fractures. Chondrocalcinosis, periarticular calcification, pseudogout, and an increased risk of atypical femoral fractures may occur (24,27-29).

### Nosology

The classification of HPP is based on the age of the patient at the onset of the first signs or symptoms of the disease, as follows: perinatal lethal, benign prenatal, infantile, childhood, adult, and odontohypophosphatasia (9). Most clinical forms of the disease share the same signs and symptoms, with overlapping clinical manifestations that vary in intensity. Although serious complications occur predominantly in childhood, they can occur at any age (24).

In the perinatal form of HPP, the most severe form of the disease, signs are present before or at birth. This form of the disease manifests with diffuse bone demineralization, pulmonary hypoplasia, respiratory failure requiring mechanical ventilation, hypotonia, and vitamin B6-dependent seizures. The infant also presents inability to gain weight and irritability. The perinatal form of HPP has a poor prognosis and is lethal if left untreated (30-32).

**Figure 2:**
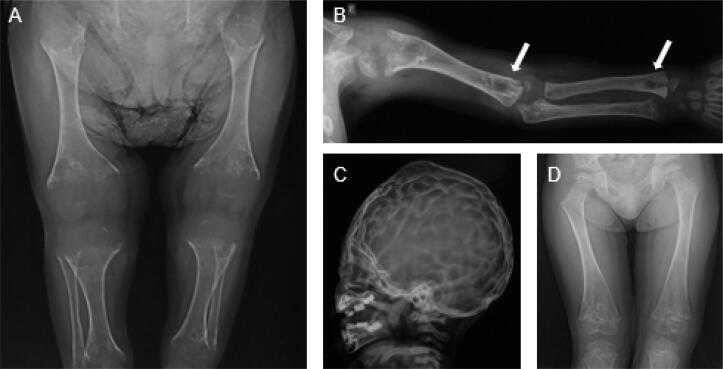
Male patient with a benign prenatal form of hypophosphatasia. He had presented manifestations before birth but was only diagnosed with the disease at the age of 1 year and 8 months. He was started on asfotase alfa at the age of 2 years and 10 months. (**A**) Age 1 year and 2 months: upper limb radiograph showing diffuse bone hypomineralization, cupping, fraying, and widening metaphyses, thin cortical bone, and diaphyseal shortening. (**B**) Metaphyseal radiolucent “tongues” (arrows). (**C**) “Beaten copper” sign. (**D**) Age 5 years, after 2 years of treatment, showing improved bone mineralization and formation, cupping, fraying and widening metaphyses, and radiolucent “tongues.” The long bone became better defined, and the lytic and sclerotic metaphyseal areas resolved. Adapted from: Arch Endocrinol Metab. 2021;64(5):623-9.

The prenatal benign form of HPP is detected before birth by gestational ultrasonography, which may show shortening of long bones and hypomineralization (Figure 2). The clinical manifestations vary between those of the severe infantile form and odontohypophosphatasia (30).

In infantile HPP, symptoms develop in the first 6 months of life. Children with this form of the disease present with failure to thrive, hyporexia, reduced muscle strength, delayed motor development, and signs of rickets. Increased intracranial pressure with papilledema, hypercalcemia, hypercalciuria, and nephrocalcinosis may occur. The occurrence of chest deformity, fractured ribs, and tracheomalacia contribute to pneumonia. Vitamin B6-dependent seizures are also frequent (30,31).

Childhood HPP manifests after the age of 6 months and can range from mild to severe (9). Patients with childhood HPP have bone changes typical of rickets, such as increased costochondral junction, increased joint spaces, fringed metaphysis, and limb deformities. Short stature and muscle weakness contribute to delayed motor development. Symptoms vary greatly in this form of HPP, which has no single characteristic clinical feature (9,33).

The adult form of HPP is characterized by an increased risk of fractures (including pseudofractures), tooth loss, muscle weakness, and chronic pain (34). In addition, excess PPi is deposited in joints and cartilage in the form of calcium pyrophosphate, leading to chondrocalcinosis (35). Ectopic calcification of varying severity is a frequent manifestation of the adult form of HPP (36).

Odontohypophosphatasia, the most common and mild form of the disease, manifests at any age with early tooth loss but no bone abnormalities. Tooth loss occurs without pain or blood and with the root intact, and is caused by abnormalities affecting the cementum, a thin bone layer that supports the dental arch (37). Early tooth loss can occur in all forms of HPP and is an important sign of the disease in affected children (38).

### Diagnosis

The diagnosis of HPP is established by clinical manifestations, radiological findings, and laboratory tests. The laboratory tests that corroborate the diagnosis of HPP are low age- and sex-adjusted serum ALP activity and elevated levels of plasma vitamin B6 and urinary PEA (15). Although ALP levels cannot be used to classify HPP subtypes yet, they seem to vary according to the severity of the disease and are higher in cases of odontohypophosphatasia and lower in more severe cases of the disease (39).

Although a well-described disease, HPP is still poorly recognized, and the lower limit level of ALP is often neglected (40). Some results are even reported without mentioning the low reference value for the patient's age. In individuals with low ALP levels, the diagnosis is complemented by high vitamin B6 levels (15). In questionable cases, measurement of PPi and PEA may help establish the diagnosis, although these tests are not commercially available in Brazil (41).

In children with reduced ALP levels, an algorithm has been suggested for the diagnosis of HPP. The first step is to assess ALP levels for age and sex and determine if the blood sample was drawn appropriately, without citrate or EDTA contamination. Subsequently, other causes of hypophosphatasemia should be excluded, such as the use of corticosteroids, antiresorptive drugs (bisphosphonates and denosumab) and clofibrate, presence of Cushing's syndrome, massive blood transfusion, milk-alkali syndrome, vitamin D intoxication, vitamin C deficiency, zinc or magnesium deficiency, hypothyroidism, celiac disease, malnutrition, osteogenesis imperfecta, and Wilson disease (24,41,42). In the following steps, the algorithm takes into account clinical symptoms and radiological signs (knee and skull X-rays) suggestive of the disease. Elevated vitamin B6 and urinary PEA levels can contribute to the diagnosis, although the latter test is not commercially available in Brazil (41).

Conditions that increase ALP levels can mask the diagnosis of HPP, including hepatobiliary disease, orthopedic surgery, and fractures (43).

Genetic testing helps to confirm the diagnosis of HPP and is important for family counseling. Family members should also be evaluated, as *de novo* mutations of the *TNSALP* gene are rare (44). The active search may unmask mild symptoms in affected family members. Children with a positive family history of HPP and those with symptoms suggestive of HPP or heterozygous carriers who have normal ALP should be reassessed, as tests can change through life (26). Special attention should be dedicated to first-degree relatives of patients with HPP.

**Figure 3:**
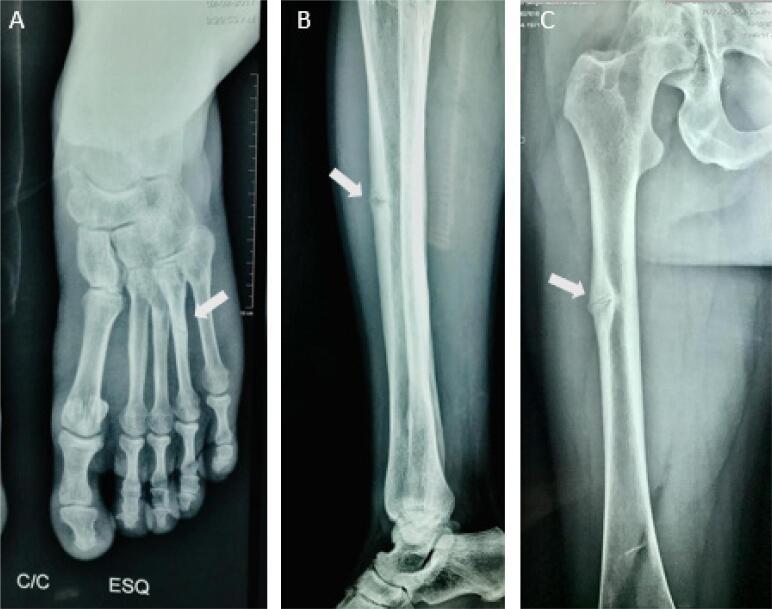
Male patient with hypophosphatasia since childhood, which was mistreated as rickets. The diagnosis of hypophosphatasia was established at the age of 49 years. The patient had short stature, skull deformity, and pseudofractures (arrows) in the left foot (**A**), right femur (**B**), and right tibia (**C**), which compromised his gait and significantly impaired his quality of life and ability to work. After starting treatment with asfotase alfa, the patient's clinical manifestations improved.

Radiographic abnormalities of HPP predominate in the metaphyses, which are flared, irregular, and fringed (Figure 2A). Other radiographic abnormalities include thinning of diaphyses, diffuse bone hypomineralization, long bone deformities, pseudofractures, delay in postfracture healing, and craniosynostosis features (14,45). Pseudofractures, a localized flaw in bone tissue due to hypomineralization, present with a radiologic appearance similar to that of a fracture (Figure 3). A characteristic finding of HPP is the presence of radiolucent “tongues,” which are areas of poorly mineralized bone in metaphyses (Figure 2B). Additionally, skull radiographs may show a beaten copper appearance (Figure 2C) (27).

Iliac crest biopsy of non-demineralized bone can show impaired calcium distribution within the mineralized bone matrix, excess osteoid, altered trabecular microarchitecture, and increased number of osteoblasts. These findings may not be present in mild cases (46).

### Enzyme replacement therapy

The treatment of HPP is aimed at improving symptoms and reducing complications. The involvement of a multidisciplinary team with experience in metabolic bone diseases is fundamental (24). Depending on the patient's manifestations, the following measures may be part of treatment: respiratory support, pyridoxine for seizures, dietary calcium restriction, nonsteroidal anti-inflammatory drugs (*NSAIDs*) for pain management, surgery for craniosynostosis, immobilization of fractures, motor and respiratory physiotherapy, and dental hygiene and care (47,48). Vitamin D levels must be maintained within normal levels to prevent the development of secondary hyperparathyroidism; excessive doses of vitamin D should be avoided, as they worsen hypercalcemia (48). Bisphosphonates, the main medications used to treat osteoporosis, are PPi analogues, and their use can worsen mineralization even further and increase the incidence of fractures (49,50).

The current treatment for moderate to severe forms of HPP is enzyme replacement therapy. Asfotase alfa (Strensiq; Alexion Pharmaceuticals, Inc.) was approved in 2015 for treatment of the pediatric form of HPP in many countries and, in 2017, was approved in Brazil by the health surveillance agency (Anvisa). Asfotase alfa is a modified copy of the human ALP enzyme, produced through recombinant DNA. It is composed of an active domain of TNSALP, an Fc fragment of IgG1, which prolongs the medication's half-life in circulation, and a deca-aspartate domain for binding of asfotase alfa to hydroxyapatite (43).

The first study showing the benefit of treatment with asfotase alfa was published in 2012 and evaluated the treatment in 11 children with perinatal and infant HPP. Clinical and radiological improvements were evidenced after 24 to 48 weeks of treatment (30). Asfotase alfa improved overall survival, height Z-score, muscle weakness, bone pain, bone mineralization, respiratory function, radiographic signs of rickets, and quality of life (Figure 2D) (30,51). The patients showed a sustained improvement in bone mineralization and growth and in respiratory, cognitive, and motor function (52).

Historical data have shown that the survival of patients without treatment is 42% and 27% at 1 and 5 years, respectively; with asfotase alfa, these rates have increased to 95% and 84%, respectively (53). One of the greatest benefits of asfotase alfa for patients with the severe form of the disease was an improvement in respiratory function. Additionally, improved bone mineralization reduces chest deformity and instability and, consequently, the occurrence of episodes of pneumonia and dyspnea (53,54). However, treatment interruption rapidly worsens the signs and symptoms of rickets (55,56).

The use of asfotase alfa to treat the pediatric form of HPP has benefits even when the medication is started later in life, even in adulthood, improving physical functioning and quality of life (57,58). Treatment of hypophosphatasia has also shown benefits in severe cases of the adult form of the disease (59).

The most common adverse effects of treatment with asfotase alfa are injection site reactions, which are usually mild and characterized by transient erythema, pruritus, discoloration, nodules, and swelling (30). Hypocalcemia may occur at the beginning of treatment; thus, calcium levels should be monitored, and calcium supplementation started if necessary. Other adverse events are hyperphosphatemia, which is usually mild but can worsen ectopic calcifications, local lipodystrophy, and hypersensitivity reactions (18,60). Ectopic eye calcification, nephrocalcinosis, and craniosynostosis have also been described, but there is still insufficient data to confirm that these complications have occurred due to treatment, as they are also manifestations of the disease (61).

### Follow-up

A monitoring guidance for patients with HPP treated with asfotase alfa has been published by an international expert panel of physicians to guide the patients' follow-up (61). The guidance highlights the importance of monitoring, on a regular basis, the levels of ALP, calcium, phosphorus, PTH, and vitamin D, as well as kidney function. Serum levels of ALP increase with treatment but are currently not used for titration of medication doses. Still, monitoring ALP levels is important to assess the patient's adherence to treatment and the presence of neutralizing antibodies (in patients on regular medication without clinical response) (61). Regarding serum PTH levels, the initial PTH measurement is usually at the lower limit of normal, and the levels may increase with treatment. At diagnosis, phosphorus levels are frequently at or above the upper limit of normal, while calcium levels may be slightly elevated (62).

Regarding imaging tests, radiographs of knees, wrists, and chest are used for treatment monitoring (61). Kidney ultrasonography and ophthalmological evaluation should be performed for the detection of ectopic calcifications that occur in the disease. In children, craniosynostosis should be monitored, and fundoscopic examination performed to assess intracranial hypertension (24). In many cases of craniosynostosis, neurosurgical intervention is necessary.

Clinical examination should include assessment of gait, muscle strength, pain, respiratory function, dental hygiene, growth (length, height, weight, and head circumference), nutrition, and quality of life (61).

### Hypophosphatasia in adults

In adulthood, HPP presents with manifestations across different body systems, pain, and reduced quality of life, regardless of the age at which the disease first manifested (63). Adult patients with HPP may have the adult form or an undiagnosed pediatric form of the disease, but in terms of clinical manifestations, both forms show no substantial differences (64).

Adult HPP can be misdiagnosed as osteoporosis, and when treated as such, the clinical manifestations of the disease may worsen further. Of note, the use of bisphosphonates in patients with HPP increases the risk of atypical femoral fracture (48,49). The risk is greater in patients on long-term bisphosphonate use and with signs of osteomalacia (65).

When compared with patients with low bone mass and no HPP, patients with HPP tend to be younger and have more metatarsal and femoral shaft fractures and fewer vertebral fractures. Additionally, patients with HPP have a higher bone mineral density in the spine and femur than patients with low bone mass (66). Notably, chondrocalcinosis may help in the differential diagnosis between osteoporosis and HPP. Bone mineral density analyzed by dual-energy X-ray absorptiometry (DXA) is usually only slightly reduced in patients with HPP. Therefore, fracture risk should not be based on DXA parameters (67).

Teriparatide (recombinant parathyroid hormone 1-34) and anti-sclerostin monoclonal antibody have been used off-label in adults with HPP. Teriparatide shows variable effects; some patients present an increase in ALP levels and improvement in bone formation markers, bone mineral density, and fracture healing, while others do not respond to the medication (68-73). This variable effect may occur due to disease severity, noncompliance, or previous use of bisphosphonates or denosumab (69,73,74). The response to teriparatide is not sustained, and levels of ALP and bone formation markers return to baseline values during treatment or within 1 year from interruption of the medication (69,70). A study using histomorphometry has shown increased cortical bone formation after treatment with teriparatide in a patient with HPP (75). PTH 1-84 has also demonstrated improvement in pain and mobility and fracture healing during treatment in two patients (76). Since the effect of teriparatide is transient and no studies with long-term use of the medication have been conducted in this population, its greatest benefit seems to be in accelerating fracture healing and in critical situations.

The effect of treatment with anti-sclerostin monoclonal antibody has been demonstrated in one study, showing increased levels of ALP and bone formation markers (procollagen type I N-terminal propeptide [PINP], osteocalcin), improved bone mineral density, and reduced bone resorption marker C-telopeptide of type I collagen (CTX-1), albeit also with a transient effect (77). Both teriparatide and anti-sclerostin monoclonal antibody are treatment options for patients with concomitant HPP and osteoporosis and for those with atypical fractures caused by bisphosphonate use (78,79). More studies are needed to prove the real benefit of these medications and evaluate the effect of prolonged use and discontinuation in patients with HPP.

In conclusion, HPP is a heterogeneous disease with different degrees of severity that may affect all age groups. Lower limits of ALP levels, which are present in HPP, are still largely neglected, complicating the diagnosis of the disease and the establishment of its actual prevalence in the population. For proper interpretation of ALP levels, values specific to the patient's age and sex must be considered. Adequate treatment of HPP is fundamental, as medications commonly used to treat other bone diseases worsen the clinical manifestations of the disease. Asfotase alfa has changed the prognosis of the disease, which was lethal in severe cases and debilitating in many patients.
